# P-480. Improving HIV Screening Rates among US Veterans: A Quality Improvement Project

**DOI:** 10.1093/ofid/ofae631.679

**Published:** 2025-01-29

**Authors:** Khushbu Shah, Matthew Martin, Lisa Fisher, Viraj Modi, Audun J Lier

**Affiliations:** Stony Brook University Hospital, Stony Brook, New York; Stony Brook University Hospital, Stony Brook, New York; Northport VA Medical Center, Northport, New York; Northport VA Medical Center, Northport, New York; Northport VA Medical Center, Northport, New York

## Abstract

**Background:**

Fifteen percent of the 1.2 million people living with human immunodeficiency virus (HIV) in the United States are unaware of their infection, which increases risk of onward transmission and negative sequalae from untreated HIV. To improve early detection of HIV infection, the US Preventive Services Task Force recommends one-time HIV testing for all adults aged 15 to 65. The goal of this quality improvement project is to understand the rate of HIV testing in US Veterans (USV) who seek care at the Northport Veterans Affairs Medical Center (NVAMC) primary care setting and assess the efficacy of a quality improvement strategy to improve HIV screening rates.

**Table 1:**
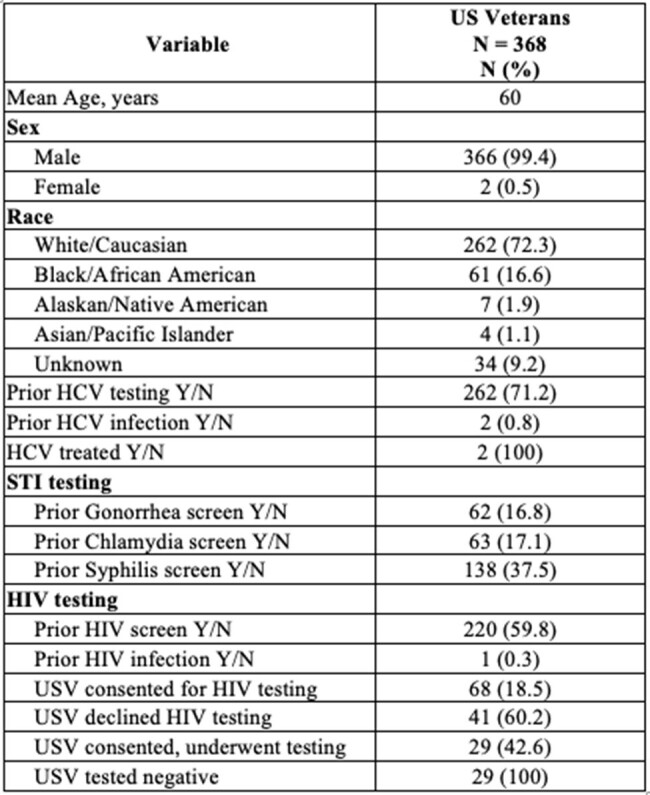
HIV, HCV, and STI screening rates, and demographic variables identified in Veterans at NVAMC primary care setting. NVAMC, Northport Veterans Affairs Medical Center; HIV, Human Immunodeficiency Virus; HCV, Hepatitis C Virus; STI, Sexually Transmitted Infections; USV, United States Veterans

**Methods:**

We conducted a prospective study of USV aged 15 to 65 from three Primary Care provider panels at NVAMC. Demographics, sexual history, injection drug use (IDU), prior hepatitis C virus (HCV), and sexually transmitted infection (STI) testing rates were obtained from chart review. USV without prior HIV screening were telephoned to obtain informed HIV testing consent and testing was ordered for USV who agreed.

**Results:**

We identified 368 USV, with mean age of 60 years. The majority of this cohort were male (n=366, 99.4%) and White (n=262, 72.3%). Seventy-five (20.4%) USV did not have sexual orientation screening charted, no USV were identified as men who have sex with men (MSM), and 4 (1.1%) USV were identified with IDU history. Two hundred twenty (59.8%) USV had received prior HIV testing. Sixty-eight (18.5%) USV with no prior testing gave consent to obtain an HIV test, 29 (42.6%) USV underwent testing and 29 (100%) tested negative for HIV. Prior HCV testing was common (n=262, 71.2%); 2 (0.8%) USV had prior HCV infection and were treated. Sixty-two (16.8%) USV received testing for gonorrhea, 63 (17.1%) for chlamydia, and 138 (37.5%) for syphilis; one USV was identified with chlamydia infection history. We identified patient barriers to HIV testing (refusal, provider outside VA system) and provider factors (testing not offered).

**Conclusion:**

Implementation of a telephone strategy improved HIV testing rates at NVAMC. HIV screening is cost effective as early identification improves survival due to linkage to care and access to treatment. Consequently, this may prevent forward transmission of HIV.

**Disclosures:**

**All Authors**: No reported disclosures

